# Periarticular myositis and muscle fibrosis are cytokine-dependent complications of inflammatory arthritis

**DOI:** 10.1172/jci.insight.179928

**Published:** 2025-03-04

**Authors:** Jessica Day, Cynthia Louis, Kristy Swiderski, Angus Stock, Huon Wong, Wentao Yao, Bonnia Liu, Suba Nadesapillai, Gordon S. Lynch, Ian P. Wicks

**Affiliations:** 1 Inflammation Division, The Walter and Eliza Hall Institute of Medical Research, Victoria, Australia.; 2 Department of Medical Biology, The University of Melbourne, Victoria, Australia.; 3 Department of Rheumatology, The Royal Melbourne Hospital, Victoria, Australia.; 4 Centre for Muscle Research, Department of Anatomy and Physiology, The University of Melbourne, Victoria.; 5 Department of Nuclear Medicine, The Royal Melbourne Hospital, Victoria, Australia.

**Keywords:** Inflammation, Muscle biology, Arthritis, Rheumatology, Skeletal muscle

## Abstract

The deleterious consequences of chronic synovitis on cartilage, tendon, and bone in rheumatoid arthritis (RA) are well described. In contrast, its effects on periarticular skeletal muscle are under-studied. Furthermore, while TNF inhibition is an effective therapy for RA synovitis, it exacerbates fibrosis in muscle injury models. We aimed to investigate whether myositis and muscle fibrosis are features of inflammatory arthritis and evaluate whether targeted RA therapies influence these disease features. Periarticular muscle was analyzed in murine models of poly- and monoarticular inflammatory arthritis: serum transfer–induced arthritis, collagen-induced arthritis, K/BxN, and antigen-induced arthritis (AIA). Periarticular myositis and an increase in muscle fibroadipocyte progenitors (FAPs) were observed in all models, despite diverse arthritogenic mechanisms. Periarticular muscle fibrosis was observed from day 15 in AIA. Neither etanercept nor baricitinib suppressed periarticular myositis or subsequent fibrosis compared to vehicle, despite reducing arthritis. Notably, etanercept failed to prevent muscle fibrosis even when initiated early, but this was not linked to increased FAP survival or collagen production. Corroborating these data, radiographic and histological analyses revealed periarticular myositis in patients with RA. We conclude that periarticular myositis and fibrosis are under-recognized features of inflammatory arthritis. Targeted RA therapies may not prevent periarticular muscle sequelae, despite controlling arthritis.

## Abstract 2

Inflammation and fibrosis in muscles surrounding joints are unrecognised complications of inflammatory arthritis. Targeted RA therapies reduce arthritis but fail to prevent muscle inflammation or fibrosis, highlighting a therapeutic gap.

## Introduction

Rheumatoid arthritis (RA) is a chronic inflammatory disease characterized by the infiltration of autoreactive immune cells into synovial joints. Research on RA has long focused on joint pathology, but there is increasing interest in the potential myopathic effects of this disease. Reduced skeletal muscle mass affects 31%–49% (1, 2) of individuals with RA and is associated with an increased risk of falls and fracture (2), impaired physical function (3, 4), and reduced quality of life (5). RA-associated myopathy has been attributed to muscle atrophy caused by disuse, systemic inflammation, and cumulative glucocorticoid exposure (6). However, the precise mechanisms underlying muscle damage in RA remain poorly understood. In particular, the possibility that periarticular muscle inflammation (myositis) and damage may occur in association with chronic synovitis, akin to that observed for local bone, cartilage, and tendons, has not been extensively studied. Histopathological studies conducted between the 1940s and 1970s identified inflammatory lesions in periarticular muscles of 25%–100% of RA cases (7–9); however, to our knowledge, contemporary literature on this phenomenon is lacking. A recent report examined patients with RA for periarticular myositis using gadolinium-enhanced MRI and determined that edema of the lumbrical muscles in the hands was present in the majority of individuals with RA (10).

The coexistence of periarticular myositis with arthritis may have clinical relevance. Chronic myositis causes muscle fibrosis, which compromises muscle function and physiology. Recent research has identified the cellular and molecular players responsible for muscle repair and how these become dysregulated during chronic muscle injury, ultimately leading to fibrosis (11). Acute muscle injury induces transient proliferation of local fibroadipocyte progenitors (FAPs), which are the main producers of ECM in skeletal muscle (12, 13). FAPs facilitate early muscle regeneration after injury (14), but timely cessation of FAP proliferation and a rapid reduction in their numbers is required to prevent muscle fibrosis. Following muscle injury, inflammatory macrophages are recruited (15) and induce FAP apoptosis, primarily through the release of tumor necrosis factor (TNF), which swiftly restores the FAP population to preinjury levels, thus preventing fibrosis (16). This phase is followed by a phenotypic switch to proreparative macrophages, which promote myofiber regeneration through the release of transforming growth factor β (TGF-β) (15).

Chronic muscle injury disrupts this sequence of events, resulting in the sustained presence of both inflammatory and proreparative macrophages. In this setting, the transition from a TNF-rich to a TGF-β–rich microenvironment is compromised, providing mixed cytokine signals to the FAP compartment. TGF-β acts as a survival signal for FAPs, promoting their differentiation into a fibrogenic phenotype (17, 18) and may block TNF-induced FAP apoptosis in vitro (16). Consequently, in chronic muscle injury, FAPs persist and undergo differentiation into collagen-producing cells, culminating in muscle fibrosis.

In this study, we provide, to our knowledge, the first evidence that periarticular skeletal muscle is inflamed in 3 well-established models of experimental arthritis (19, 20), despite diverse arthritogenic mechanisms. We confirm that periarticular myositis includes bone marrow–derived macrophages with an activated phenotype, residing in a mixed cytokine milieu. This is accompanied by persistent expansion of periarticular muscle FAPs that express abundant collagen, ultimately resulting in periarticular muscle fibrosis. We further demonstrate that treatment with etanercept, an anti-TNF agent commonly used in the treatment of RA, exacerbates periarticular muscle fibrosis, most likely by inhibiting TNF-induced apoptosis of periarticular muscle FAPs. Finally, we provide radiological and histological evidence of periarticular myositis in patients with inflammatory arthritis. This study provides insights into the mechanisms of periarticular myositis and how it contributes to disability in RA.

## Results

### Periarticular myositis is a frequent feature of experimental arthritis.

**Figure 1:**
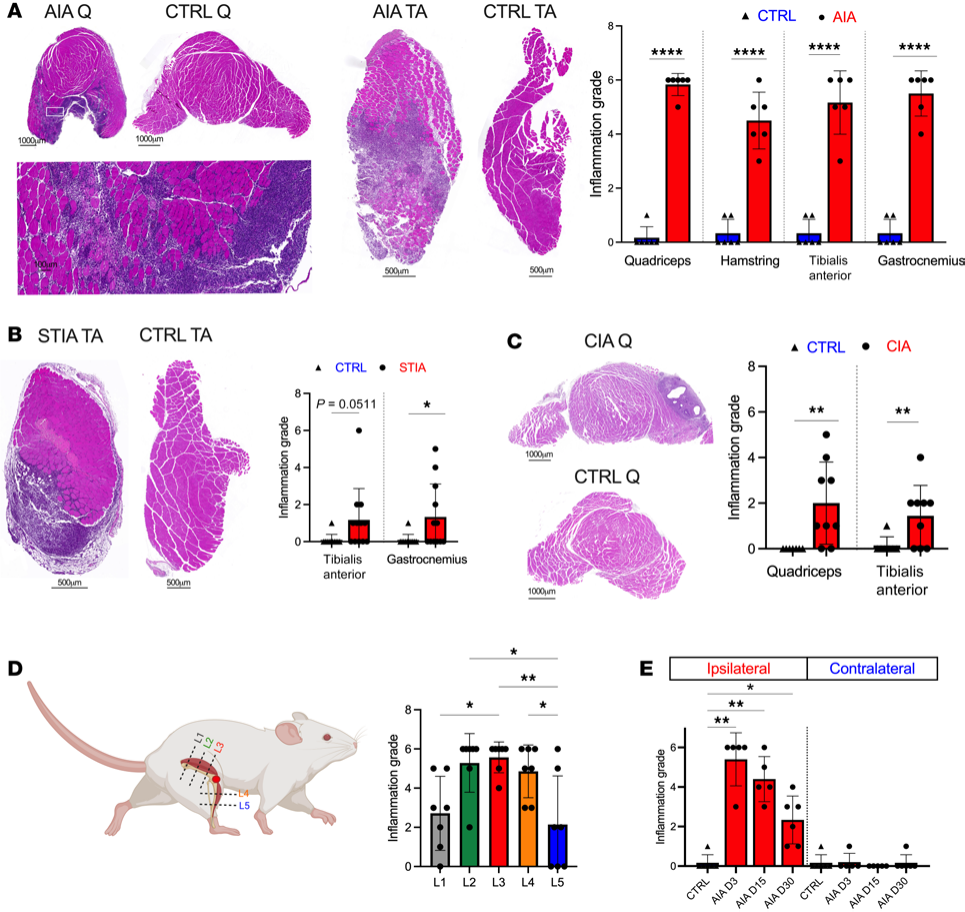
Periarticular myositis complicates multiple experimental models of inflammatory arthritis. (**A**) H&E-stained sections of periarticular quadriceps (Q), tibialis anterior (TA), hamstrings, and gastrocnemius muscles from AIA (*n* = 6) and control mice (*n* = 6) were graded for inflammation. The quadriceps grade is the mean of vastus lateralis and vastus medialis scores. Representative images are presented. High-magnification view corresponds to boxed region in the low-magnification image of the quadriceps. Data are representative of more than 3 independent experiments. (**B**) H&E-stained sections from periarticular gastrocnemius and TA muscles obtained from STIA (*n* = 12) and control (*n* = 11) mice were graded for inflammation. (**C**) H&E-stained sections from periarticular quadriceps and TA muscles from CIA (*n* = 9) and control mice (*n* = 7) were graded for inflammation. (**D**) H&E-stained sections from sequential levels of periarticular muscle in AIA mice (*n* = 7), as indicated in the schematic, were graded for inflammation. Schematic prepared with BioRender. (**E**) Ipsilateral and contralateral periarticular quadriceps muscles were obtained from AIA mice receiving a unilateral intraarticular injection of mBSA (*n* = 5–6 per time point) and graded for inflammation on day 3 (D3), D15, and D30. Mean ± SD presented. Statistical significance was determined using unpaired Student’s *t* tests (**A**–**C**), 1-way ANOVA followed by Bonferroni’s post hoc correction (**D**), or a mixed effects model followed by Šidák’s post hoc correction (**E**). For **E**, time-course comparisons were analyzed only for ipsilateral data. Scale bars: 1000 μm (low-magnification Q images, **A** and **C**), 100 μm (high-power Q image, **A**), and 500 μm (TA images, **A** and **B**). *P < 0.05; **P < 0.01; ****P < 0.0001.

To investigate whether periarticular myositis occurs in experimental arthritis, we evaluated the periarticular muscles of mice with different models of experimental arthritis — collagen-induced arthritis (CIA), antigen-induced arthritis (AIA), K/BxN, and serum transfer–induced arthritis (STIA). For models with polyarticular arthritis (CIA, K/BxN, and STIA), we examined the intrinsic muscles of the paws, distal tibialis anterior, and distal gastrocnemius. For models with knee arthritis (AIA and CIA), we examined the distal hamstrings, distal quadriceps, proximal tibialis anterior, and proximal gastrocnemius. Periarticular myositis was observed in all 4 disease models (Figure 1, A–E, and Supplemental Figure 1; supplemental material available online with this article; https://doi.org/10.1172/jci.insight.179928DS1).

To evaluate the anatomical distribution of the myositis in AIA (knee monoarthritis), we examined the quadriceps and tibialis anterior muscles at sequential levels relative to the inflamed knee. Myositis was most severe in the periarticular region (Figure 1D). A striking anatomical distribution of myositis was observed in AIA quadriceps, where muscles that abut the knee joint (vastus lateralis and vastus medialis) were profoundly inflamed, while anatomically removed muscles (rectus femoris) were comparatively spared (Figure 1A and Supplemental Figure 1A). To further confirm that myositis was secondary to local arthritis and not a systemic process, AIA was induced unilaterally, with the contralateral knee receiving an intra-articular injection of PBS. Myositis was only observed ipsilateral to the inflamed joint in these experiments (Figure 1E). An advantage of the AIA model is the synchronized, well-defined onset of monoarthritis, rendering it useful for time-course studies. Longitudinal analysis of this model revealed a gradual decline in periarticular myositis out to day 30, coinciding with the late, destructive, and less inflammatory arthritic phase of AIA (19, 21) (Figure 1E).

### Chronic macrophage infiltration contributes to a dysregulated cytokine milieu within the periarticular muscle of inflammatory arthritis.

**Figure 2:**
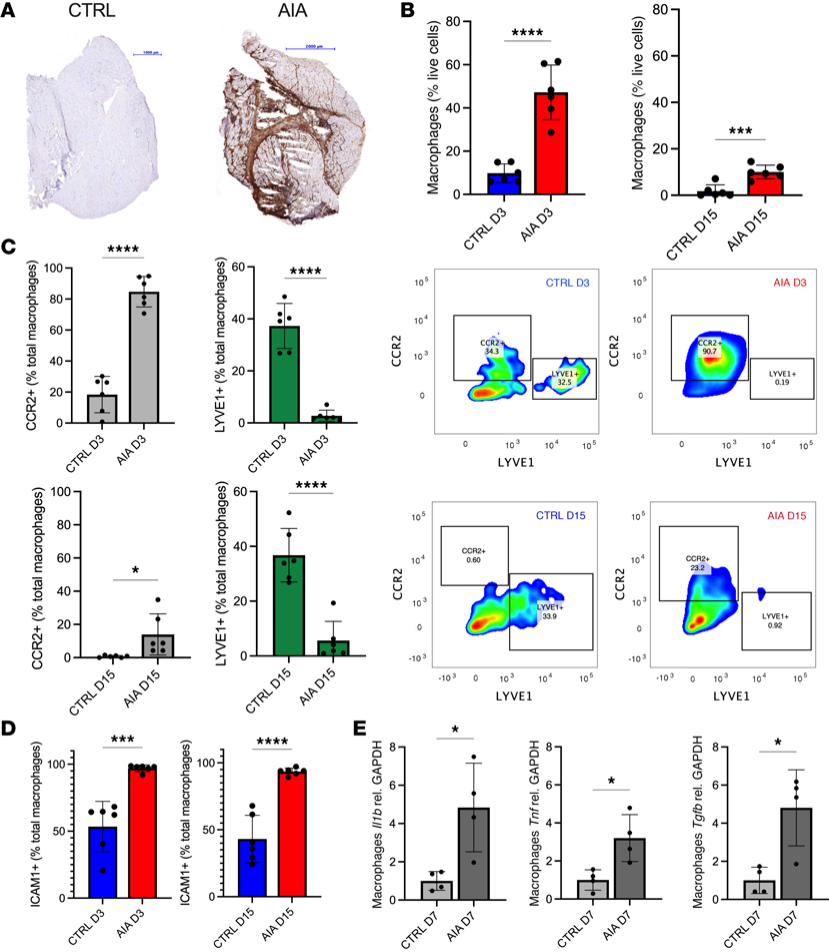
Chronic macrophage infiltration contributes to a dysregulated cytokine milieu within periarticular muscle during inflammatory arthritis. (**A**) F4/80 staining of hamstring muscles from AIA and control mice. (**B**) Flow cytometric detection of CD45^+^CD11b^+^Ly6G^–^MHC II^+^CD64^+^ macrophages in periarticular quadriceps from AIA mice (*n* = 6 per time point) versus control (*n* = 6 per time point) on day 3 (D3) and D15. Data representative of 3 independent experiments. (**C**) Macrophages from periarticular quadriceps muscles of AIA (*n* = 6 per time point) and control mice (*n* = 6 per time point) were analyzed with respect to LYVE1 and CCR2 expression using flow cytometry on D3 and D15. (**D**) Periarticular quadriceps macrophages were evaluated for intercellular adhesion molecule-1 (ICAM1) expression in AIA (*n* = 6 per time point) versus control mice (*n* = 6 per time point) on D3 and D15 using flow cytometry. (**E**) Macrophages were isolated from periarticular muscle of AIA (*n* = 15) and control mice (*n* = 15) using FACS and analyzed for IL-1β, TNF, and TGF-β gene expression by qPCR; each data point represents muscle tissue pooled from 3–4 mice. Mean ± SD presented. Statistical significance was determined using unpaired Student’s *t* tests. Scale bars: 1000 μm (CTRL) and 2000 μm (AIA). *P < 0.05; ***P < 0.001; ****P < 0.0001.

To further characterize periarticular myositis in experimental arthritis, we conducted immunohistochemical and flow cytometric analyses (Supplemental Figure 2). Given that macrophages are critical regulators of muscle regeneration following acute injury but have a profibrotic role in chronic muscle injury (16), we focused on these cells. AIA muscle featured extensive F4/80^+^ macrophage infiltration at the peak of arthritis (day 3 after arthritis induction) (Figure 2A), which decreased as joint inflammation resolved (day 15) (Supplemental Figure 3A). Flow cytometric analysis confirmed that macrophages (CD45^+^CD11b^+^Ly6G^–^MHC II^+^CD64^+^) were significantly increased in the periarticular quadriceps of AIA mice compared with controls (Figure 2B).

We next determined whether periarticular muscle macrophages in inflammatory arthritis are derived from recruited monocytes or from tissue-resident macrophages that proliferate locally. To address this question, we evaluated the expression of LYVE1, which is widely expressed by tissue-resident macrophages (22–24) and CCR2, a chemokine receptor that is expressed by short-lived, monocyte-derived macrophages (22). We found an increase in CCR2^+^ macrophages within AIA periarticular muscle in both the early (day 3) and chronic (day 15) phases of the model, which was accompanied by a striking reduction in the proportion of LYVE1^+^ macrophages (Figure 2C). This pattern was also observed in periarticular muscles from STIA and K/BxN mice (Supplemental Figure 3D). Periarticular muscle macrophages (Figure 2D and Supplemental Figure 3E) were also positive for intercellular adhesion molecule-1 (ICAM1), which is a marker of activated macrophages (25, 26) associated with efferocytosis and macrophage polarization (25, 27, 28). Thus, activated CCR2^+^ macrophages infiltrate periarticular muscles during inflammatory arthritis, and this is accompanied by a reduction in the proportion of tissue-resident macrophages.

The persistence of CCR2^+^ macrophages beyond the acute phase of the AIA model is of interest. While systemic CCR2 deletion results in muscle fibrosis after acute injury (16), inducible deletion of CCR2^+^ macrophages following the initial phase of inflammation in cardiac vasculitis models ameliorates myocardial fibrosis (22). This suggests that while CCR2^+^ macrophages may have beneficial roles in acute muscle injury, these cells might exert deleterious, profibrotic effects in the setting of chronic myositis.

Given the time-related, reciprocal effects of TNF and TGF-β in muscle repair, we examined cytokine expression by periarticular muscular macrophages (CD45^+^CD11b^+^Ly6G^–^MHC II^+^CD64^+^) in experimental arthritis. Specifically, we sorted these cells from AIA and control muscle and performed quantitative PCR. For these experiments, we examined muscle macrophages on day 7 after arthritis induction, when muscle enters a restorative phase after acute injury and the number of TNF-producing macrophages typically returns to steady state (29). However, macrophages isolated from day 7 AIA periarticular muscle had mixed cytokine expression profiles, with significantly elevated inflammatory *Tnf*, *Il1b*, and proreparative *Tgfb1* mRNA compared with macrophages from control periarticular muscle (Figure 2E). Thus, chronic macrophage infiltration contributes to a dysregulated cytokine milieu within the periarticular muscle of mice with inflammatory arthritis.

### Periarticular myositis results in fibroadipocyte expansion and muscle fibrosis in inflammatory arthritis.

**Figure 3:**
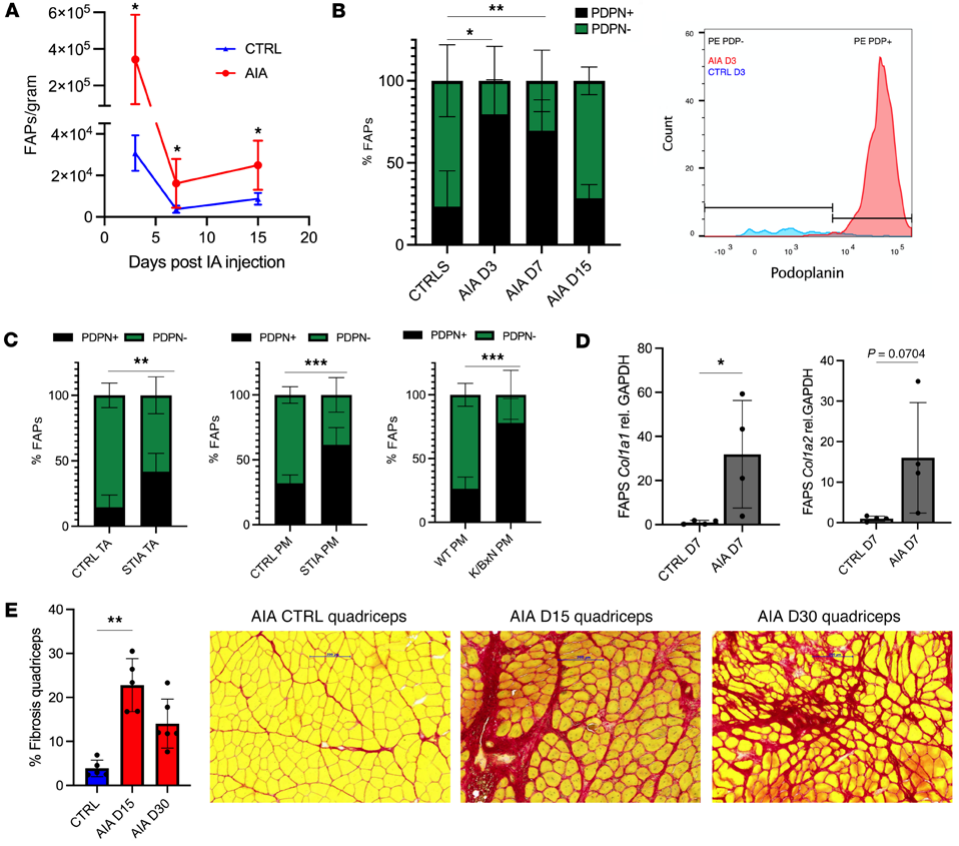
Periarticular myositis causes fibroadipocyte expansion and muscle fibrosis in inflammatory arthritis. (**A**) FAPs from periarticular quadriceps muscles of AIA mice (*n* = 6–7 per time point) and controls (*n* = 6 per time point) were enumerated using flow cytometry at 3 disease time points. IA, intra-articular. (**B**) The proportion of podoplanin^+^ (PDPN^+^) FAPs within the periarticular quadriceps of AIA and control mice was evaluated using flow cytometry at 3 time points (*n* = 6–7 per group). A representative FACS plot demonstrating PDPN expression by FAPs isolated from AIA and control mice on day 3 after injection (D3) is presented. (**C**) The proportion of PDPN^+^ FAPs within periarticular muscle of STIA mice (*n* = 7), K/BxN mice (*n* = 5), and relevant controls (*n* = 6 per group) were analyzed using flow cytometry. (**D**) FAPs were isolated from periarticular quadriceps muscles of AIA (*n* = 15) and control mice (*n* = 15) using FACS and analyzed for collagen type I gene expression. Each data point represents muscles pooled from 3–4 mice. (**E**) The extent of fibrosis within the periarticular quadriceps muscles of AIA (*n* = 5–6 per time point) and control mice on D15 and D30 was quantified using ImageJ software and color masking to detect the percentage of red or green within the entire Sirius red– or Masson’s trichrome–stained section, respectively. High-power images of representative Sirius red–stained sections are presented. Scale bars: 200 μm. D15 data representative of 3 independent experiments. Statistical significance was determined using a mixed effects model followed by Šidák’s post hoc correction (**A**, **B**, and **E**) or unpaired Student’s *t* tests (**C** and **D**). Mean ± SD presented. TA, tibialis anterior; PM, intrinsic paw muscles. *P < 0.05; **P < 0.01; ***P < 0.001.

An undesirable consequence of chronic muscle injury is the persistence of FAPs, which produce multiple ECM proteins, such as collagens, proteoglycans, laminins, and fibronectin (30), leading to muscle fibrosis. Using flow cytometry, we confirmed an expansion of CD45^–^CD31^–^Sca1^+^PDGFRα^+^ FAPs (11) (Supplemental Figure 2) in the periarticular muscle of AIA mice (Figure 3A), which persisted beyond days 5–9 (Figure 3A), when these cells have typically returned to steady-state levels following acute injury (12, 16). Podoplanin (PDPN) is a marker of activated FAPs that migrate from subcutaneous adipose tissue into muscle during the very early phase after injury, before returning to basal levels by day 3 after injury (31). We observed an increased proportion of PDPN^+^ FAPs in the periarticular muscle of AIA mice, which persisted beyond day 3 and normalized by day 15 (Figure 3B). An increased proportion of PDPN^+^ FAPs was also observed in the periarticular muscle of the STIA and K/BxN models (Figure 3C). We hypothesized that the prolonged persistence of activated FAPs, coupled with production of TGF-β from local intramuscular macrophages, might cause these cells to acquire a fibrogenic phenotype. Indeed, FAPs isolated from the periarticular quadriceps muscles of AIA mice 7 days after induction of arthritis expressed higher levels of collagen type 1 (*Col1a1* and *Col1a2*) mRNA than FAPs isolated from control periarticular muscle (Figure 3D). Sirius red staining confirmed greater interstitial collagen deposition in the periarticular muscles of AIA mice on days 15 and 30 compared with control mice (Figure 3E).

### Targeted RA therapies ameliorate joint pathology but not periarticular muscle fibrosis in experimental arthritis.

**Figure 4:**
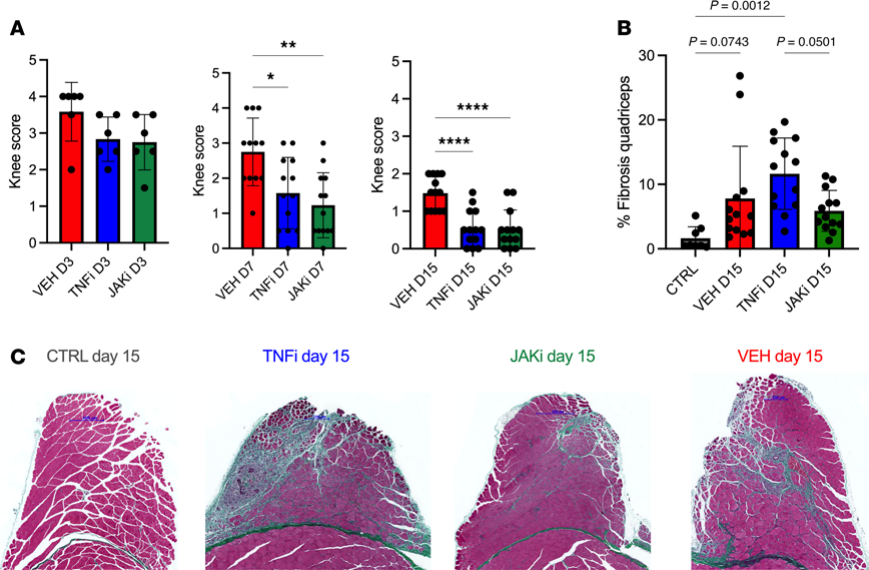
TNF inhibition ameliorates joint pathology but exacerbates periarticular muscle fibrosis in inflammatory arthritis. (**A**) AIA mice received second daily doses of etanercept (TNG inhibitor, TNFi), baricitinib (JAKi), or vehicle (VEH) from day 1 (D1) after arthritis induction. Knee arthritis in AIA mice was assessed blinded using a visual grading system (*n* = 6–13 per group). (**B**) The extent of fibrosis within the periarticular quadriceps muscles of AIA (*n* = 13 per treatment group) and control mice (*n* = 8) on D15 was quantified using Masson’s trichrome staining. (**C**) Representative images of Masson’s trichrome–stained quadriceps on D15. Scale bars: 500 μm (CTRL, JAKi) and 200 μm (TNFi, VEH). Statistical significance was determined using 1-way ANOVA followed by Tukey’s post hoc correction (**A**–**C**). Mean ± SD presented. *P < 0.05; **P < 0.01; ****P < 0.0001.

Given the critical role of TNF in inducing FAP clearance via apoptosis following muscle injury (16), we hypothesized that TNF inhibition might exacerbate periarticular muscle fibrosis in experimental inflammatory arthritis. We therefore treated AIA mice with etanercept, an anti-TNF agent used in clinical practice that also neutralizes murine TNF (32). We compared the effects of etanercept to baricitinib, a Janus kinase 1/2 inhibitor used to treat RA. As expected, administration of these agents consistently reduced arthritis (Figure 4A). However, the effect of these agents on periarticular muscle inflammation was modest, with no significant differences in the proportion of periarticular macrophage infiltration observed between vehicle-, etanercept-, or baricitinib-treated mice as assessed by FACS analysis (Supplemental Figure 4A). The macrophage phenotype also remained similar between the 3 groups, with equivalent proportions of ICAM1^+^ and CCR2^+^ periarticular muscle macrophages (Supplemental Figure 4B).

Neither etanercept nor baricitinib effectively suppressed muscle fibrosis compared with vehicle in the context of arthritis (Figure 4, B and C). Etanercept treatment was associated with a significant increase in periarticular muscle fibrosis on day 15 compared with control non-arthritic mice (Figure 4B). A significant increase in fibrosis compared with non-arthritic controls was also observed in vehicle- or baricitinib-treated mice when treatment was delayed until day 4 after arthritis induction (Supplemental Figure 4C).

**Figure 5:**
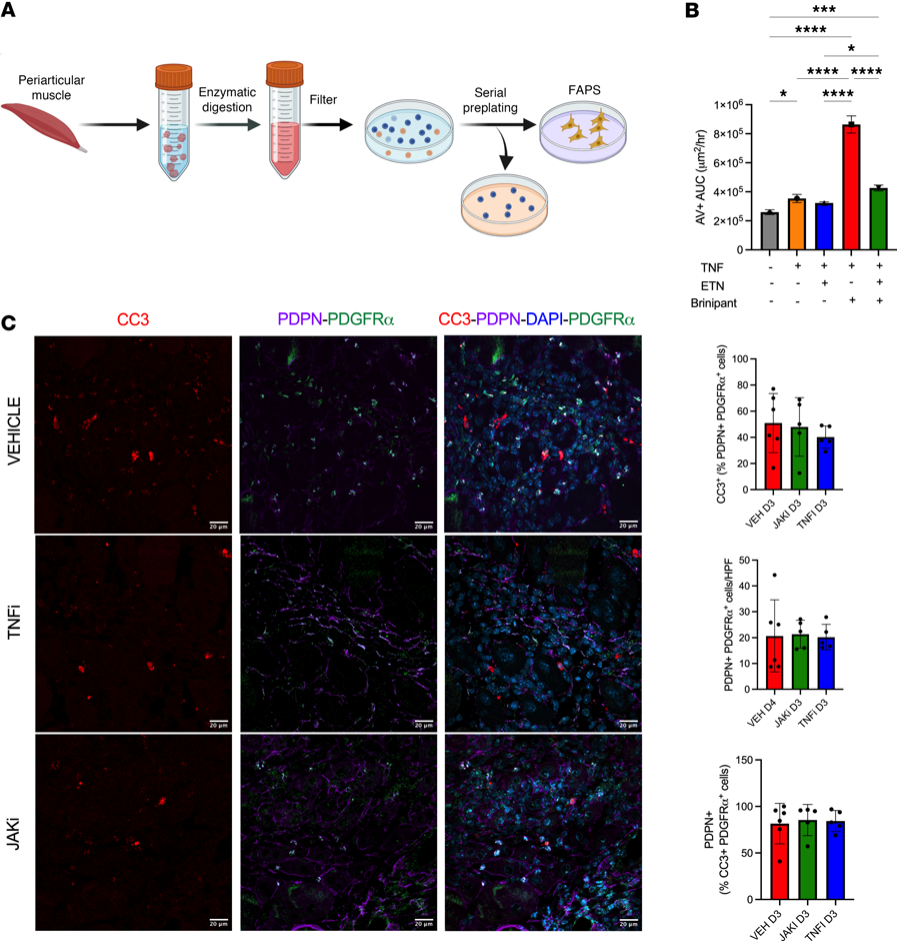
TNF inhibition alters cell death of periarticular muscle FAPs in vitro but not in vivo. (**A**) Primary muscle fibroblast cell cultures were generated from digested and filtered periarticular muscle using a preplating technique. (**B**) Periarticular muscle fibroblast cultures were treated with TNF, birinapant, etanercept (ETN), or combinations thereof (*n* = 4 per group). Cells were stained with annexin V (AV) and imaged hourly over 24 hours using the IncuCyte platform. Data presented as AV^+^ area under the curve (AUC). (**C**) Periarticular quadriceps muscles from AIA mice treated with etanercept (TNFi, *n* = 5), baricitinib (JAKi, *n* = 5) or vehicle (*n* = 6) were collected on day 3 after arthritis induction, stained for cleaved caspase 3 (CC3, to detect apoptotic cells), PDGFRα, and podoplanin (PDPN, to detect activated FAPs), and analyzed using immunofluorescence confocal microscopy. The number of cells per high-power field was quantified and relevant proportions calculated. Scale bars: 20 μm. Isotype control staining is shown in Supplemental Figure 5. Statistical significance was determined using 1-way ANOVA followed by Tukey’s post hoc correction (**B** and **C**). Mean ± SD presented. *P < 0.05; ***P < 0.001; ****P < 0.0001.

Previous research has indicated that TNF inhibition promotes fibrosis by reducing TNF-induced apoptosis of FAPs in inflamed muscle, leading us to question whether this mechanism could contribute to fibrosis in the etanercept-treated AIA mice. To explore this, we isolated fibroblasts using a preplating method (33) from the periarticular muscle of healthy mice (Figure 5A). Muscle fibroblasts isolated in this way are phenotypically and biochemically equivalent to FAPs (34). Treatment of periarticular muscle fibroblasts with TNF only marginally increased apoptosis, as determined by annexin V staining (Figure 5B). This finding contrasts with previous data suggesting TNF substantially increases apoptosis in muscle fibroblasts in vitro (16). However, our findings align with the understanding that TNF activates NF-κB signaling and cellular inhibitors of apoptosis (IAPs), thereby promoting cell survival and proliferation. TNF cytotoxicity requires inactivation of intracellular cell death checkpoints, such as IAPs (35). Thus, to sensitize cells to death, we cotreated periarticular muscle fibroblasts with TNF and birinapant, a second mitochondrial activator of caspases (SMAC) mimetic compound that downregulates IAPs. As expected, the combination of TNF and birinapant markedly increased apoptosis, which was partially rescued by etanercept (Figure 5B). These results suggest that TNF induces apoptosis only in periarticular fibroblasts that are already primed to undergo cell death.

To determine whether TNF inhibition affects cell death of periarticular muscle FAPs in vivo, we examined muscle from AIA mice treated with vehicle, etanercept, or baricitinib. For this analysis, we evaluated the number and proportion of cells staining positive for cleaved caspase 3 (CC3, a marker of apoptosis), PDPN (a marker of activated FAPs), and platelet-derived growth factor α (PDGFRα) using immunofluorescence. Staining of day 3 AIA muscle revealed that the proportions of PDPN^+^PDGFRα^+^ cells undergoing apoptosis (i.e., CC3^+^) in the periarticular region were comparable across the 3 treatment groups (Figure 5C). Similarly, the total number of PDPN^+^PDGFRα^+^ cells per high-power field was equivalent among the treatment groups (Figure 5C). Almost all apoptotic FAPs in the periarticular region were PDPN^+^, suggesting that activated FAPs are more prone to death; this finding was also consistent across all groups (Figure 5C). We also determined whether collagen production by periarticular FAPs differed between treatment groups using qPCR and found that it did not (Supplemental Figure 4D). Collectively, these data suggest that although TNF inhibition rescues FAPs undergoing apoptosis in vitro, this mechanism is not the major contributor to periarticular muscle fibrosis in vivo. Instead, other factors, such as inadequate suppression of periarticular myositis, may play a more prominent role in driving fibrosis in this context.

### Preliminary observations of periarticular myositis in patients with inflammatory arthritis.

**Figure 6:**
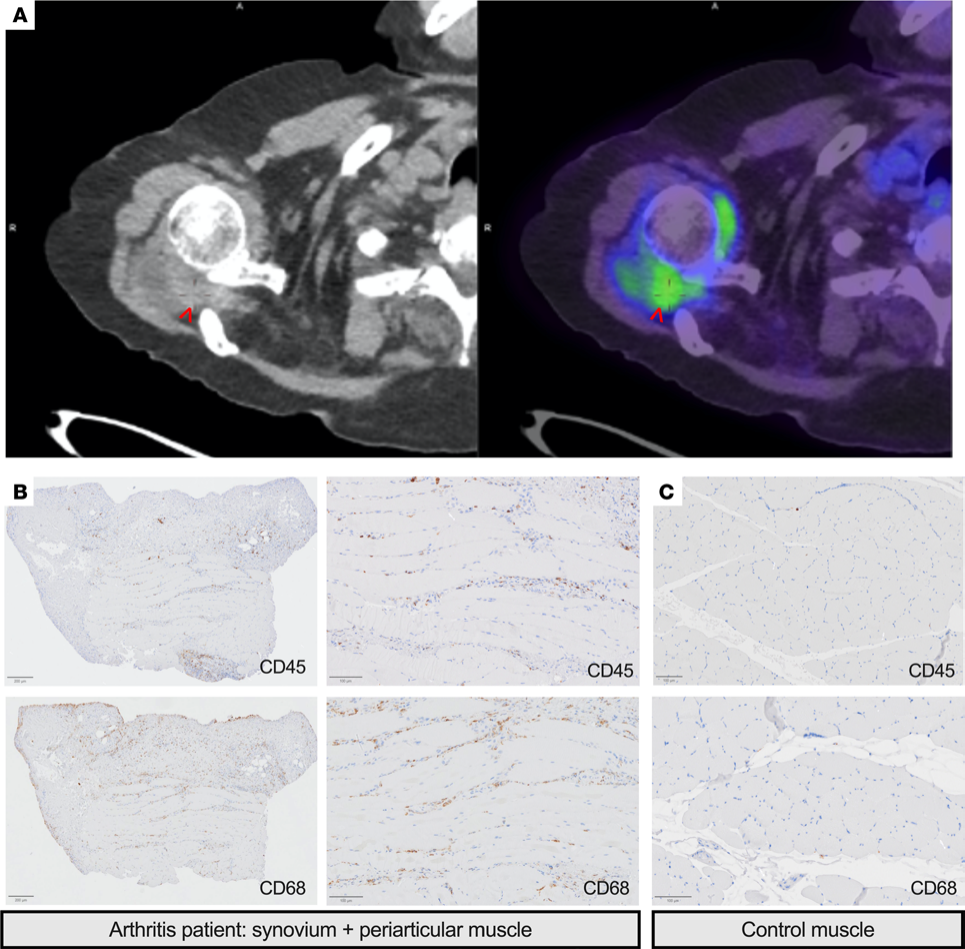
Periarticular myositis in patients with inflammatory arthritis. (**A**) PET/CT imaging of a 78-year-old woman with rheumatoid arthritis and lung cancer. The patient was on methotrexate (10 mg weekly) and prednisolone (15 mg daily) at the time of the scan. Imaging demonstrates shoulder synovitis with associated periarticular muscle avidity (arrow). (**B**) Sections of knee synovium and periarticular muscle from a 19-year-old man with symmetrical, polyarticular inflammatory arthritis were stained for CD45 and CD68 using immunohistochemistry. Low-power image depicts synovium and periarticular muscle. High-power image depicts periarticular muscle. (**C**) Quadriceps muscle from a patient with multiple sclerosis was stained for CD45 and CD68 using immunohistochemistry. Scale bars: 200 μm (left panel) and 100 μm (middle and right panels).

Previous research has demonstrated lumbrical muscle edema in greater than 70% of patients with active RA using gadolinium-enhanced MRI (10). We hypothesized that large joint periarticular myositis may be detectable via positron emission tomography–computed tomography (PET/CT), as there is increasing interest in using this modality to profile inflammatory myopathy (36). We retrospectively identified 5 patients with RA who underwent ^18^F-fluorodeoxyglucose (FDG) PET/CT (Supplemental Table 3). Two of the 5 RA patients had definitive evidence of abnormal periarticular muscle avidity in the shoulders (Figure 6A), despite immunosuppression (Supplemental Table 3). Although these observations are based on a small sample size, they provide insights into potential periarticular muscle involvement in RA and complement previous reports (10).

To assess for histological evidence of periarticular myositis in the setting of inflammatory arthritis, we searched the Melbourne Health Anatomical Pathology database for synovial joint specimens with periarticular muscle attached. We identified one such sample from a 19-year-old man with bilateral, symmetrical polyarthritis who was seronegative for rheumatoid factor and anti–cyclic citrullinated peptide antibodies. He underwent a joint aspirate and synovial biopsy. Synovial histopathology revealed mild active chronic synovitis composed of lymphocytes and histocytes, with associated synovial cell hyperplasia. Synovial fluid and tissue cultures revealed no causative organism, and he was ultimately diagnosed with a non-infectious inflammatory polyarthritis. The synovial biopsy also captured periarticular muscle (Figure 6B), which demonstrated inflammation and CD68^+^ macrophage infiltration. In comparison, few inflammatory cells were present in histologically normal quadriceps muscle obtained from a patient with multiple sclerosis (Figure 6C). This case, along with historical literature, provides proof of principle that local muscle can be infiltrated by inflammatory cells in the context of sterile synovitis.

## Discussion

It is well recognized that individuals with RA experience muscle weakness, which adversely affects function and quality of life. Despite the classical effects of chronic synovitis on local cartilage, tendon, and bone, there has been a surprising lack of attention paid to periarticular muscle in RA. Most of the contemporary literature examining muscle involvement in RA has centered on the concept of RA-associated sarcopenia, defined as a progressive and generalized skeletal muscle disorder characterized by the accelerated loss of muscle mass and function (37). This phenomenon has been attributed to the muscle-wasting effects of systemic cytokine dysregulation, with mechanistic studies describing excessive muscle catabolism driven by cytokine-induced activation of the ubiquitin proteasome system (38–40).

Periarticular myositis has been reported in RA (7–9), although these findings predate the biologic and “treat to target” era. Recent investigations evaluating periarticular muscle in RA are scarce. Some studies have demonstrated reduced tissue oxygen levels (41) and radiological evidence of inflammation (10) in the intrinsic hand muscles of RA patients compared with controls. Molecular profiling of vastus lateralis muscle from individuals with RA revealed greater concentrations of IL-6 compared with controls, although concentrations of IL-1β and TNF were equivalent (42). However, our data suggest that profiling mid-belly muscle may profoundly underestimate the degree of muscle damage that accumulates in RA. To our knowledge, this is the first comprehensive demonstration that periarticular myositis is a consistent and striking feature across multiple well-established models of experimental poly- and monoarticular arthritis. We also provide preliminary radiographic and histological evidence of periarticular myositis in patients with inflammatory arthritis. Whether this occurs as a secondary effect of cytokine spread from the joint, activating tissue-resident cells and inducing further immune cell infiltration, or whether muscle represents another target of autoimmunity in RA requires further consideration. The ubiquitous nature of periarticular myositis across diverse models of experimental arthritis makes the former more likely. Importantly, the persistence of periarticular myositis despite improvements in joint inflammation with TNF or JAK inhibition raises the possibility that, once initiated, this process may be self-perpetuating or poorly responsive to these therapies.

Persistent myositis leads to fibrosis and permanent muscle damage. In this study, we demonstrate excessive accumulation of FAPs and increased TGF-β production from macrophages in the periarticular muscles of mice with chronic arthritis. This environment strongly favors fibrogenesis, and indeed, we document excessive collagen deposition within the periarticular muscles over time. Our study clarifies existing evidence examining the role of TNF in limiting the expansion of FAPs, and hence preventing pathological matrix production (16). We extend previous findings by demonstrating that FAPs from periarticular muscles undergo TNF-induced apoptosis only when intracellular IAPs are simultaneously inhibited; FAPs treated with TNF alone are relatively protected from cell death. Notably, endogenous IAP antagonists are upregulated by cellular stress (43, 44) and it is plausible that FAPs in the inflamed periarticular environment increase their expression of these proapoptotic proteins, rendering them susceptible to TNF-induced death.

These observations led us to consider whether inhibiting TNF signaling might paradoxically promote fibrosis, by allowing the survival of FAPs under cellular stress. However, our in vivo data did not support increased FAP survival due to TNF inhibition. In fact, neither TNF inhibition nor JAK inhibition suppressed periarticular myositis or fibrosis compared to vehicle in the context of arthritis. These findings are clinically relevant, as agents targeting TNF and the JAK/STAT pathway are commonly used in the treatment of RA. While concerns have been raised about the profibrotic potential of TNF depletion (16, 45, 46), TNF signaling has also been implicated in inflammation-induced fibrosis (47). Although TNF inhibition did not prevent periarticular muscle fibrosis, our data do not indicate a direct profibrotic effect. Notably, TNF inhibition was not linked with increased numbers or prolonged survival of FAPs in the periarticular muscle, nor with elevated collagen production by FAPs. Overall, our findings suggest that neither TNF inhibition nor JAK inhibition ameliorate periarticular myositis or prevent its fibrotic sequelae, suggesting the involvement of other cytokine pathways, or perhaps metabolic reprogramming of FAPs.

This study has limitations. We evaluated murine models of RA, which may not precisely reflect the human situation. However, we validated our findings in 4 distinct, well-characterized experimental models with different arthritogenic mechanisms, lending credibility to the results. Further mechanistic studies aimed at deciphering the role of cytokines and cellular components contributing to periarticular myositis in these models are clearly required. However, systemic or tissue-specific genetic deletion of key inflammatory proteins may ameliorate arthritis and confound evaluation of the associated periarticular myositis. Finally, our human studies were limited and retrospective. Although FDG PET/CT has some limitations for imaging periarticular soft-tissue structures, our preliminary findings suggest that there is extension of inflammation beyond the synovium and adjacent musculotendinous junction to involve muscle itself. These observations align with previous reports of radiographic enhancement within the periarticular lumbrical muscles in RA (10) and warrant further investigation. Our future studies will build upon these initial analyses with prospectively collected samples and a consistent approach to imaging.

In summary, we describe an aspect of disease pathogenesis in RA that we contend has been overlooked, with the potential for considerable clinical and functional impact. The periarticular skeletal muscles warrant recognition as an integral component of the synovial joint complex and an extra-articular site of inflammation. Future studies should systematically evaluate the periarticular muscles in RA and seek to better understand the pathogenesis of periarticular myositis, which may not be prevented by existing therapies.

## Methods

### Sex as a biological variable.

Our study examined both male and female animals, and we did not observe any sex differences in the measured endpoints.

### Mice.

C57BL/6 and K/BxN mice were purchased from The Jackson Laboratory and bred and maintained at The Walter and Eliza Hall Institute (WEHI, Parkville, Australia) under standard conditions. Both male and female mice 6–12 weeks of age were used for all experiments.

### Induction of arthritis and therapeutic agents.

For AIA (48), 8- to 12-week old C57BL/6 mice were immunized intradermally with methylated bovine serum albumin (mBSA) (2 mg/mL; Sigma-Aldrich) emulsified in an equal volume of complete Freund’s adjuvant (CFA) containing 5 mg/mL heat-killed *Mycobacterium* *tuberculosis* H37RA (Difco) on day –7. Arthritis was induced on day 0 by injection of 200 μg mBSA in 10 μL of 0.9% weight/volume PBS into the knee. Control mice received intradermal injections of CFA alone, followed by intra-articular mBSA, unless stated otherwise. Mice were euthanized on day 7 and a clinical severity score was assigned to each knee in a blinded manner, based on the degree of swelling and erythema: grade 0, no swelling; grade 1, mild swelling and erythema localized within the knee joint; grade 2, moderate swelling and erythema localized within the knee joint; grade 3, severe swelling in knee and surrounding muscle; grade 4, severe knee swelling with increased vascularity and joint rigidity.

Intraperitoneal injections of etanercept or PBS were commenced on day 1 or day 4 after intra-articular injection and repeated second daily for 3–15 days. Baricitinib or vehicle was administered via oral gavage on day 1 or day 4 after intra-articular injection. Mice (*n* = 7–10/group) were randomly enrolled into treatment and control groups.

For STIA (49), serum was obtained from individual K/BxN (20) mice following the onset of arthritis and then pooled. C57BL/6 mice (8–12 weeks old) received intraperitoneal injections of 100 μL serum from K/BxN mice on day 0 (*n* = 5–8/group). STIA mice were harvested on day 7, coinciding with peak arthritis.

For CIA (50), C57BL/6 (51) mice were immunized intradermally with type II chicken collagen (2 mg/mL; Sigma-Aldrich) emulsified in an equal volume of CFA on day 0 and day 21; controls received CFA only (*n* = 4–6/group). CIA mice were assessed clinically for polyarthritis severity using a well-established scoring system (20) from day 21 and harvested on day 31.

### Quantitative PCR.

For quantitative real-time PCR, FAPs and macrophages from periarticular quadriceps were sorted on a FACSAria Fusion cell sorter (BD Biosciences). Total RNA was extracted from cells using an ISOLATE II RNA Mini Kit (Bioline) and reverse transcribed into cDNA using SuperScript III Reverse Transcriptase (Invitrogen) and Oligo(dT) primers (Promega). Quantitative real-time PCR was performed using Fast SYBR Green Master Mix (Thermo Fisher Scientific) on a QuantStudio 12K PCR system (Thermo Fisher Scientific) using primers shown in Supplemental Table 1. Gene expression was normalized to *Gapdh* (Δ cycle threshold [Ct]), and values expressed as target gene mRNA level relative to *Gapdh* (2^−ΔCt^). The ΔCt data were normalized to the control group such that the mean expression of control samples was set to 1.00, facilitating the interpretation of effect sizes.

### Histology and immunofluorescence.

Murine skeletal muscle was dissected tendon to tendon and divided into periarticular (the third of muscle closest to the inflamed joint) and non-periarticular samples. Muscle was fixed in 10% neutral buffered formalin for 24 hours and then processed into paraffin blocks. Serial 10-μm-thick muscle transverse sections were prepared and mounted on coated slides. For histological analyses, sections were stained with H&E (to visualize cellular infiltration), Masson’s trichrome (MT), or Sirius red (SR) to visualize collagen deposition. To quantify fibrosis, digital images of the entire MT- or SR-stained muscle section underwent color masking (ImageJ, NIH). Green (MT) or red (SR) staining (signifying collagen) as a percentage of the whole muscle cross-sectional area was calculated. For quantification of inflammation, H&E-stained sections were scored in a blinded fashion using a well-established grading scale (0, no inflammation; 6, >100 muscle fibres surrounded or invaded by mononuclear cells) (52).

For immunofluorescence, 10-μm-thick muscle sections underwent heat-induced epitope retrieval in EDTA buffer followed by blocking with serum (Jackson Immunoresearch), BSA (Sigma-Aldrich), and Protein Block solution (Dako). Sections were stained with primary antibodies (Supplemental Table 2), followed by detection with fluorochrome-conjugated secondary antibodies. Nuclei were stained with DAPI. Consecutive images of inflamed muscle were captured using a Zeiss LSM 980 confocal microscope and analyzed with ImageJ software. ImageJ Cell Counter was used for cell quantification.

For human tissue, formalin-fixed, paraffin-embedded samples of muscle and synovium were obtained from the Royal Melbourne Hospital Anatomical Pathology Department. Samples were identified by searching the Anatomical Pathology database for relevant terms (“muscle,” “synovitis,” “arthritis”). Paraffin sections (7 μm) were dewaxed and subjected to citrate antigen retrieval. Sections were blocked with serum, BSA, and Protein Block before staining with primary antibodies (Supplemental Table 2). After washing, slides were incubated with a commercial detection system consisting of a universal secondary antibody formulation conjugated to HRP (Nichirei-Histofine). After washing, slides were incubated with chromogen/substrate (ImmPACT DAB, Vector Laboratories), rinsed, and counterstained with hematoxylin. Relevant clinical data were extracted from the medical records.

### Flow cytometry.

Periarticular mouse muscles were dissected, minced, and digested in type IV collagenase (1 mg/mL, Worthington) with Dispase (0.1 mg/mL, Worthington). Single-cell suspensions were stained with cocktails of directly conjugated antibodies against CD45, CD11b, Ly6C, Ly6G, CD64, ICAM1, MHC II, LYVE1, CCR2, CD31, Sca1, PDGFRα, PDPN, and α-7 integrin (Supplemental Table 2 and Supplemental Figure 2). Counting beads (Miltenyi Biotec) were used for cell enumeration. Samples were acquired on a FACSymphony and analyzed with FlowJo 10.10.0 software (both BD Biosciences).

### IncuCyte analysis.

For primary muscle fibroblast cell cultures, muscle from C57BL/6 mice was digested in type II collagenase (1 mg/mL, Worthington) with Dispase (0.1 mg/mL, Worthington), filtered, and plated. Sequential rounds of preplating were performed to isolate fibroblasts from satellite cells (33). This technique exploits the differential adhesion of fibroblasts to plastic compared with satellite cells, which remain in suspension (53). Primary fibroblasts were plated at subconfluence in 48-well tissue culture plates in Dulbecco’s modified Eagle’s medium containing 10% fetal bovine serum and antibiotics. After 18–24 hours in culture, nonadherent cells were removed, and media supplemented with TNF (20 ng/mL), etanercept (1 μg/mL), birinapant (1 μM), or a combination thereof, plus annexin V–FITC and PI. Cells were imaged within 10 minutes of treatment using phase contrast, and red and green channels in the IncuCyte Live Cell Analysis platform. Cells were kept within the IncuCyte for 24 hours with 5% CO_2_ and 37°C climate control. Nine image sets per well were taken every hour and each condition was run in quadruplicate. Apoptotic cells were quantified by analysis of the area (μm^2^/image) of annexin V–positive cells.

### FDG PET/CT analyses.

For imaging studies, FDG PET/CT scans of patients with active RA were independently reviewed by 2 physicians specialized in both rheumatology and nuclear medicine. Relevant scans performed between 2018 and 2023 were identified by searching radiology databases for the following terms within the body of the image report: “rheumatoid,” “arthritis,” “muscle,” and “myositis.” Images were reviewed for evidence of synovitis and focally increased muscle FDG avidity adjacent to the joint capsule. Increased muscle FDG avidity was distinguished from adjacent joint effusions on the basis of radiodensity as measured in Hounsfield units. Diffusely increased muscle FDG avidity within an entire muscle group was considered to reflect physiological muscle activation rather than inflammatory change. Clinical data were extracted from the medical records.

### Statistics.

Comparisons between 3 or more groups were performed using 1-way ANOVA with Tukey’s post hoc correction for multiple comparisons. Two-group comparisons were performed using unpaired, 2-tailed Student’s *t* tests. Time-course data were analyzed using a mixed effects model followed by Šidák’s post hoc correction for multiple comparisons. All analyses were performed using Prism version 9.5.1 (GraphPad). Data are presented as mean ± SD unless stated otherwise. A *P* value of less than 0.05 was considered statistically significant.

### Study approval.

All procedures involving mice were approved by the WEHI Animal Ethics Committee. Profiling of human tissue sections was approved by the Melbourne Health Human Research Ethics Committee (no. 2010.293). Retrospective analysis of FDG PET/CT images was approved by the Melbourne Hospital Research and Governance Office (no. QA2023137).

### Data availability.

All supporting data for this study are provided within the main text and supplemental materials. Individual data points from graphs in both the main text and supplemental materials are available in the Supporting Data Values file. Additional raw data can be requested from the corresponding author. Raw data related to human participants will be provided after deidentification and in compliance with applicable privacy laws and data protection regulations.

## Author contributions

IPW and JD conceptualized the study with input from KS and GSL. Mouse experiments were designed by JD with input from CL, KS, and AS. Mouse experiments were performed by JD, CL, AS, HW, and WY. Mouse data were acquired and analyzed by JD, HW, and WY. Experiments using patient samples were performed by JD. FDG PET/CT images were analyzed by BL and SN and relevant clinical data were collected by JD. The manuscript was written by JD with input from all authors. JD and CL contributed equally to the mouse component of this study; however, JD performed additional work involving human specimens and wrote the first manuscript draft, and hence she is listed first.

## Supplementary Material

Supplemental data

Supporting data values

## Version 1 03/04/2025

In-Press Preview

## Version 2 04/08/2025

Electronic publication

## References

[1] Li TH (2021). The prevalence and risk factors of sarcopenia in rheumatoid arthritis patients: a systematic review and meta-regression analysis. Semin Arthritis Rheum.

[2] Torii M (2019). Prevalence and factors associated with sarcopenia in patients with rheumatoid arthritis. Mod Rheumatol.

[3] Kramer HR (2012). Muscle density in rheumatoid arthritis: associations with disease features and functional outcomes. Arthritis Rheum.

[4] Baker JF (2017). Assessment of muscle mass relative to fat mass and associations with physical functioning in rheumatoid arthritis. Rheumatology (Oxford).

[5] Fukuda W (2013). Low body mass index is associated with impaired quality of life in patients with rheumatoid arthritis. Int J Rheum Dis.

[6] Bennett JL (2023). Rheumatoid sarcopenia: loss of skeletal muscle strength and mass in rheumatoid arthritis. Nat Rev Rheumatol.

[7] Kestler OC (1949). Histopathology of the intrinsic muscles of the hand in rheumatoid arthritis. Ann Rheum Dis.

[8] Magyar E (1977). Muscle changes in rheumatoid arthritis. A review of the literature with a study of 100 cases. Virchows Arch A Pathol Anat Histol.

[9] Riley M, Harrison SH (1968). Interosseous muscle biopsy during hand surgery for rheumatoid arthritis. Br J Plast Surg.

[10] Akkaya Z (2023). Lumbrical muscle enhancement on MRI and its association with rheumatoid arthritis. Skeletal Radiol.

[11] Giuliani G (2022). Signaling pathways regulating the fate of fibro/adipogenic progenitors (FAPs) in skeletal muscle regeneration and disease. FEBS J.

[12] Joe AW (2010). Muscle injury activates resident fibro/adipogenic progenitors that facilitate myogenesis. Nat Cell Biol.

[13] Uezumi A (2010). Mesenchymal progenitors distinct from satellite cells contribute to ectopic fat cell formation in skeletal muscle. Nat Cell Biol.

[14] Heredia JE (2013). Type 2 innate signals stimulate fibro/adipogenic progenitors to facilitate muscle regeneration. Cell.

[15] Arnold L (2007). Inflammatory monocytes recruited after skeletal muscle injury switch into antiinflammatory macrophages to support myogenesis. J Exp Med.

[16] Lemos DR (2015). Nilotinib reduces muscle fibrosis in chronic muscle injury by promoting TNF-mediated apoptosis of fibro/adipogenic progenitors. Nat Med.

[17] Contreras O (2019). Cross-talk between TGF-β and PDGFRα signaling pathways regulates the fate of stromal fibro-adipogenic progenitors. J Cell Sci.

[18] Uezumi A (2011). Fibrosis and adipogenesis originate from a common mesenchymal progenitor in skeletal muscle. J Cell Sci.

[19] Jones GW (2018). In vivo models for inflammatory arthritis. Methods Mol Biol.

[20] Monach PA (2008). The K/BxN arthritis model. Curr Protoc Immunol.

[21] Nissler K (2004). Anti-CD4 monoclonal antibody treatment in acute and early chronic antigen induced arthritis: influence on macrophage activation. Ann Rheum Dis.

[22] Stock AT (2019). The selective expansion and targeted accumulation of bone marrow-derived macrophages drive cardiac vasculitis. J Immunol.

[23] Dick SA (2022). Three tissue resident macrophage subsets coexist across organs with conserved origins and life cycles. Sci Immunol.

[24] Krasniewski LK (2022). Single-cell analysis of skeletal muscle macrophages reveals age-associated functional subpopulations. Elife.

[25] Hubbard AK, Giardina C (2000). Regulation of ICAM-1 expression in mouse macrophages. Inflammation.

[26] Bernatchez SF (1997). Expression of intercellular adhesion molecule-1 on macrophages in vitro as a marker of activation. Biomaterials.

[27] Dalal PJ, Sumagin R (2020). Emerging functions of ICAM-1 in macrophage efferocytosis and wound healing. J Cell Immunol.

[28] Gu W (2017). ICAM-1 regulates macrophage polarization by suppressing MCP-1 expression via miR-124 upregulation. Oncotarget.

[29] Chazaud B (2020). Inflammation and skeletal muscle regeneration: leave it to the macrophages!. Trends Immunol.

[30] Negroni E (2022). Muscle fibro-adipogenic progenitors from a single-cell perspective: focus on their “virtual” secretome. Front Cell Dev Biol.

[31] Sastourne-Arrey Q (2023). Adipose tissue is a source of regenerative cells that augment the repair of skeletal muscle after injury. Nat Commun.

[32] Kruglov AA, Nedospasov SA (2012). Regulation of immune responses by prostaglandin E2. J Immunol.

[33] Hindi L (2017). Isolation, culturing, and differentiation of primary myoblasts from skeletal muscle of adult mice. Bio Protoc.

[34] Contreras O (2019). Adherent muscle connective tissue fibroblasts are phenotypically and biochemically equivalent to stromal fibro/adipogenic progenitors. Matrix Biol Plus.

[35] Huyghe J (2023). Cell death checkpoints in the TNF pathway. Trends Immunol.

[36] Bentick G (2022). Defining the clinical utility of PET or PET-CT in idiopathic inflammatory myopathies: a systematic literature review. Semin Arthritis Rheum.

[37] Cruz-Jentoft AJ, Sayer AA (2019). Sarcopenia. Lancet.

[38] Castillero E (2009). IGF-I system, atrogenes and myogenic regulatory factors in arthritis induced muscle wasting. Mol Cell Endocrinol.

[39] Little RD (2017). Compensatory anabolic signaling in the sarcopenia of experimental chronic arthritis. Sci Rep.

[40] Li J (2020). TNF receptor-associated factor 6 mediates TNFα-induced skeletal muscle atrophy in mice during aging. J Bone Miner Res.

[41] Akhavani MA (2011). Muscle hypoxia in rheumatoid hands: does it play a role in ulnar drift?. J Hand Surg Am.

[42] Huffman KM (2017). Molecular alterations in skeletal muscle in rheumatoid arthritis are related to disease activity, physical inactivity, and disability. Arthritis Res Ther.

[43] Du C (2000). Smac, a mitochondrial protein that promotes cytochrome c-dependent caspase activation by eliminating IAP inhibition. Cell.

[44] Verhagen AM (2000). Identification of DIABLO, a mammalian protein that promotes apoptosis by binding to and antagonizing IAP proteins. Cell.

[45] Ramos-Casals M (2011). Pulmonary disorders induced by monoclonal antibodies in patients with rheumatologic autoimmune diseases. Am J Med.

[46] Wen Y (2020). TNF-α in T lymphocytes attenuates renal injury and fibrosis during nephrotoxic nephritis. Am J Physiol Renal Physiol.

[47] Verjee LS (2013). Unraveling the signaling pathways promoting fibrosis in Dupuytren’s disease reveals TNF as a therapeutic target. Proc Natl Acad Sci U S A.

[48] Staite ND (1989). The effects of sensitization protocols on arthritic responses in antigen-induced arthritis. Agents Actions.

[49] Christensen AD (2016). K/BxN serum-transfer arthritis as a model for human inflammatory arthritis. Front Immunol.

[50] Brand DD (2007). Collagen-induced arthritis. Nat Protoc.

[51] Campbell IK (2000). Collagen-induced arthritis in C57BL/6 (H-2b) mice: new insights into an important disease model of rheumatoid arthritis. Eur J Immunol.

[52] Kamiya M (2022). Targeting necroptosis in muscle fibers ameliorates inflammatory myopathies. Nat Commun.

[53] Rando TA, Blau HM (1994). Primary mouse myoblast purification, characterization, and transplantation for cell-mediated gene therapy. J Cell Biol.

