# Supplementary material for: Periarticular myositis and muscle fibrosis are cytokine-dependent complications of inflammatory arthritis
Source: JCI Insight. 2025 Mar 4;10(7):e179928. doi: 10.1172/jci.insight.179928 (PMC11981620; doi:10.1172/jci.insight.179928)
Supplement: Supplemental data [file jciinsight-10-179928-s093.pdf]

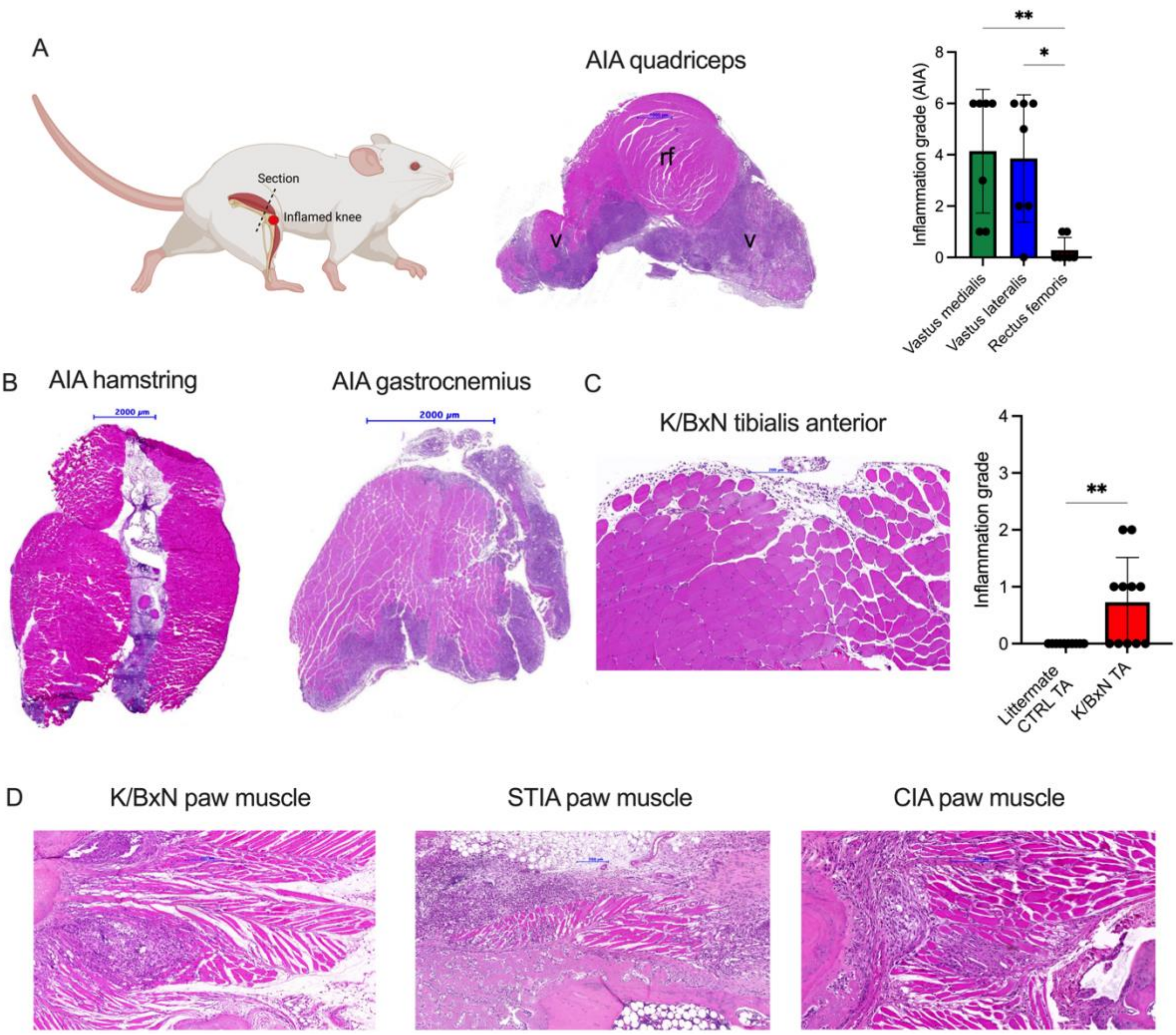

Supplementary Figure 1. (A) H&E-stained sections of periarticular quadriceps obtained from AIA mice (*n* = 7) were graded for inflammation. The vastus medialis (vm), vastus lateralis (vl) and rectus femoris (rf) muscles are annotated. Schematic demonstrates the anatomical level of muscle collected and analysed (prepared with biorender). Scale bar: 1000μm. (B) Representative H&E images demonstrating periarticular myositis in AIA hamstrings (scale bar: 2000μm) and AIA gastrocnemius muscles (scale bar: 2000μm). (C) H&E-stained sections of tibialis anterior (TA) muscles from K/BxN mice (*n* = 11) and littermate controls (*n* = 10) were graded for inflammation. Representative image presented. Scale bar: 200μm. (D) Representative H&E-stained sections demonstrating inflammation of the intrinsic paw muscles in the K/BxN, STIA and CIA models. Scale bars: 200μm.

Mean ± SD presented. * $p < 0.05$; ** $p < 0.01$.

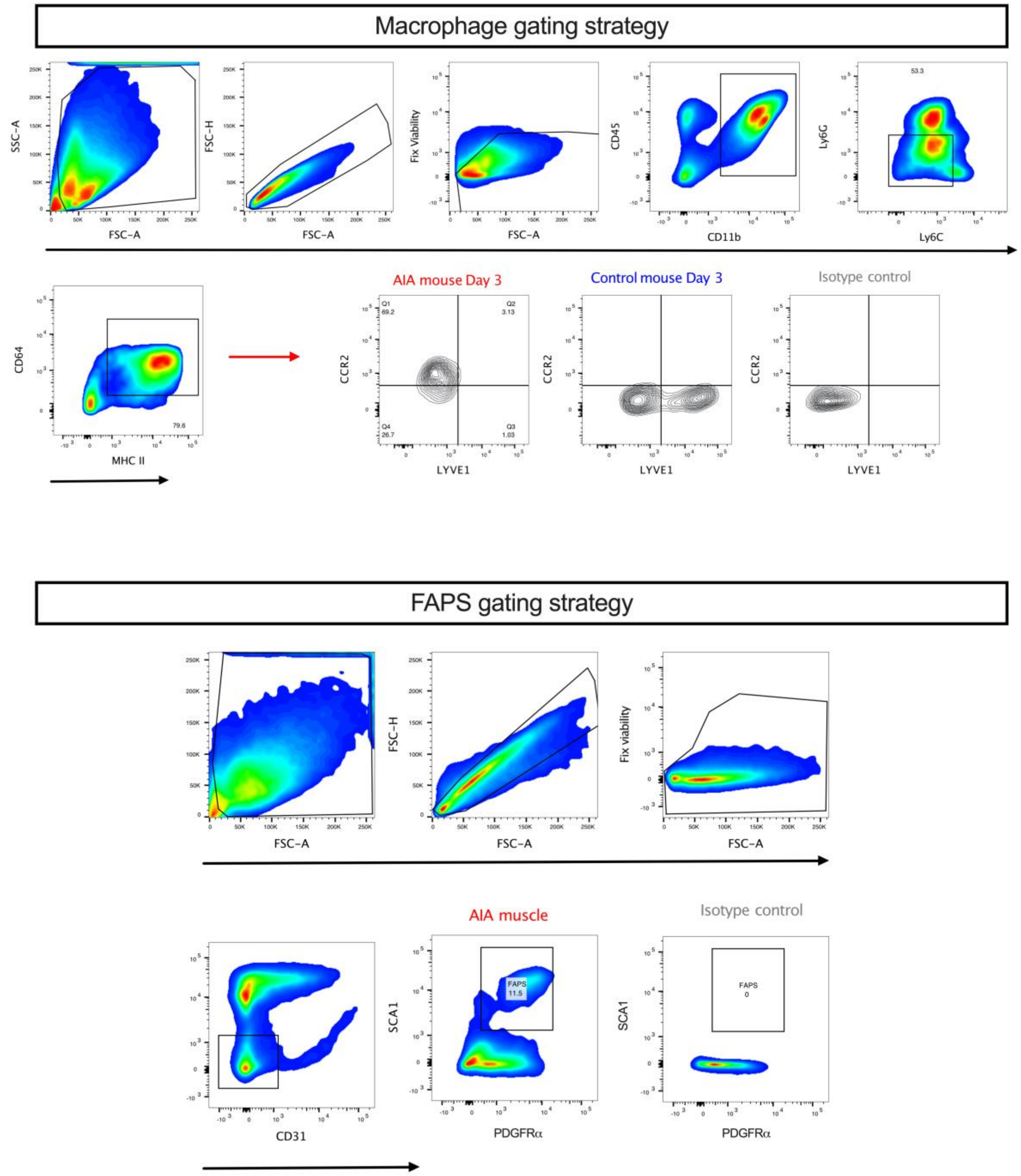

Supplementary Figure 2. Gating strategy for flow cytometric analyses.

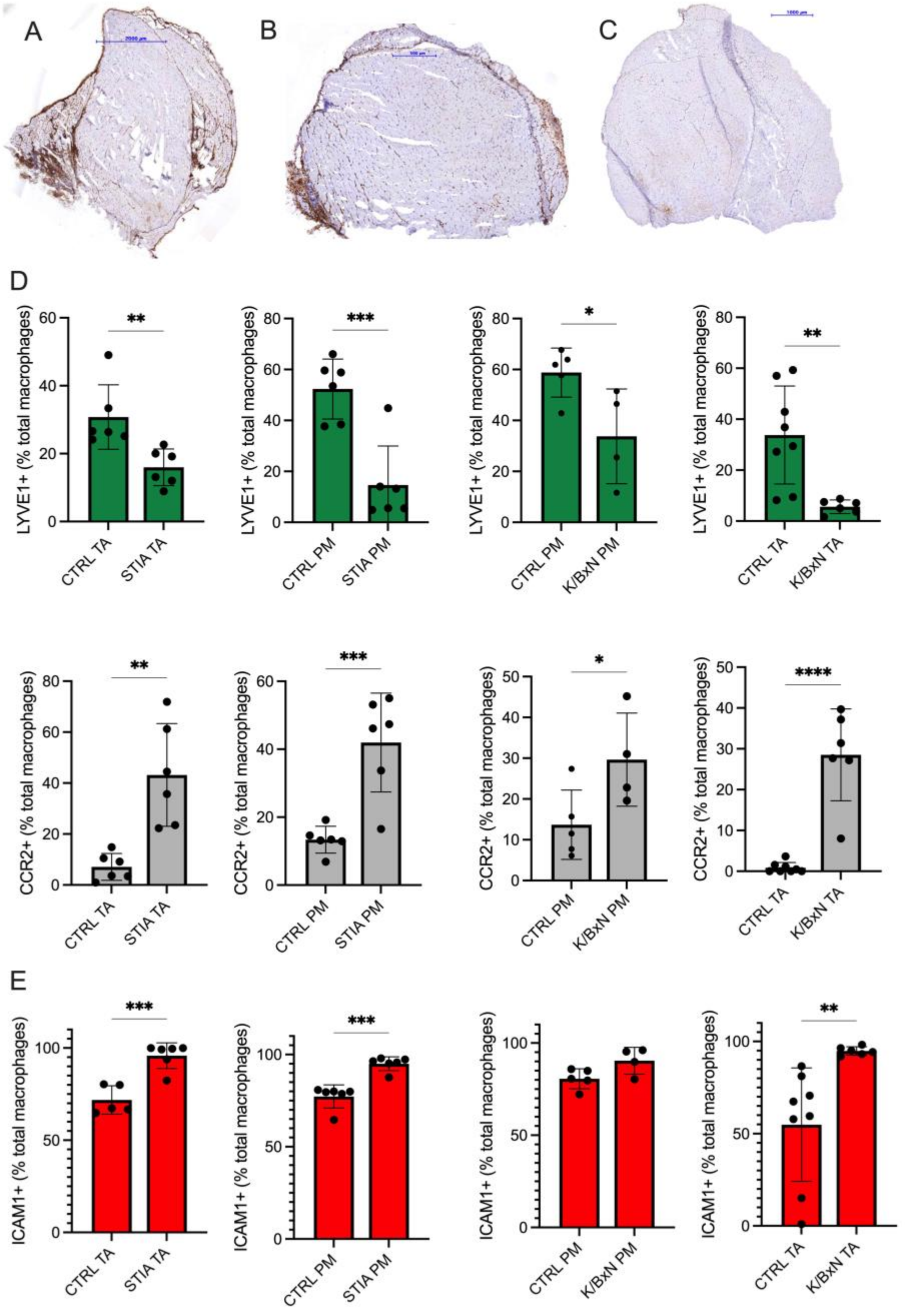

Supplementary Figure 3. F4/80 staining of quadriceps muscles from (A) an AIA mouse at day 3 (scale bar: 2000μm), (B) an AIA mouse at day 15 (scale bar: 500μm), and (C) a control mouse at day 3 (scale bar: 1000μm). (D) Macrophages from periarticular muscle (tibialis anterior, TA; intrinsic paw muscles, PM) from STIA and K/BxN mice were analysed for CCR2 and LYVE1 expression using flow cytometry (*n* = 4-8 per group). (E) Macrophages from periarticular muscles were also analysed for intercellular adhesion molecule-1 (ICAM1) expression using flow cytometry. Mean ± SD presented. * $p < 0.05$; ** $p < 0.01$; *** $p < 0.001$; **** $p < 0.0001$.

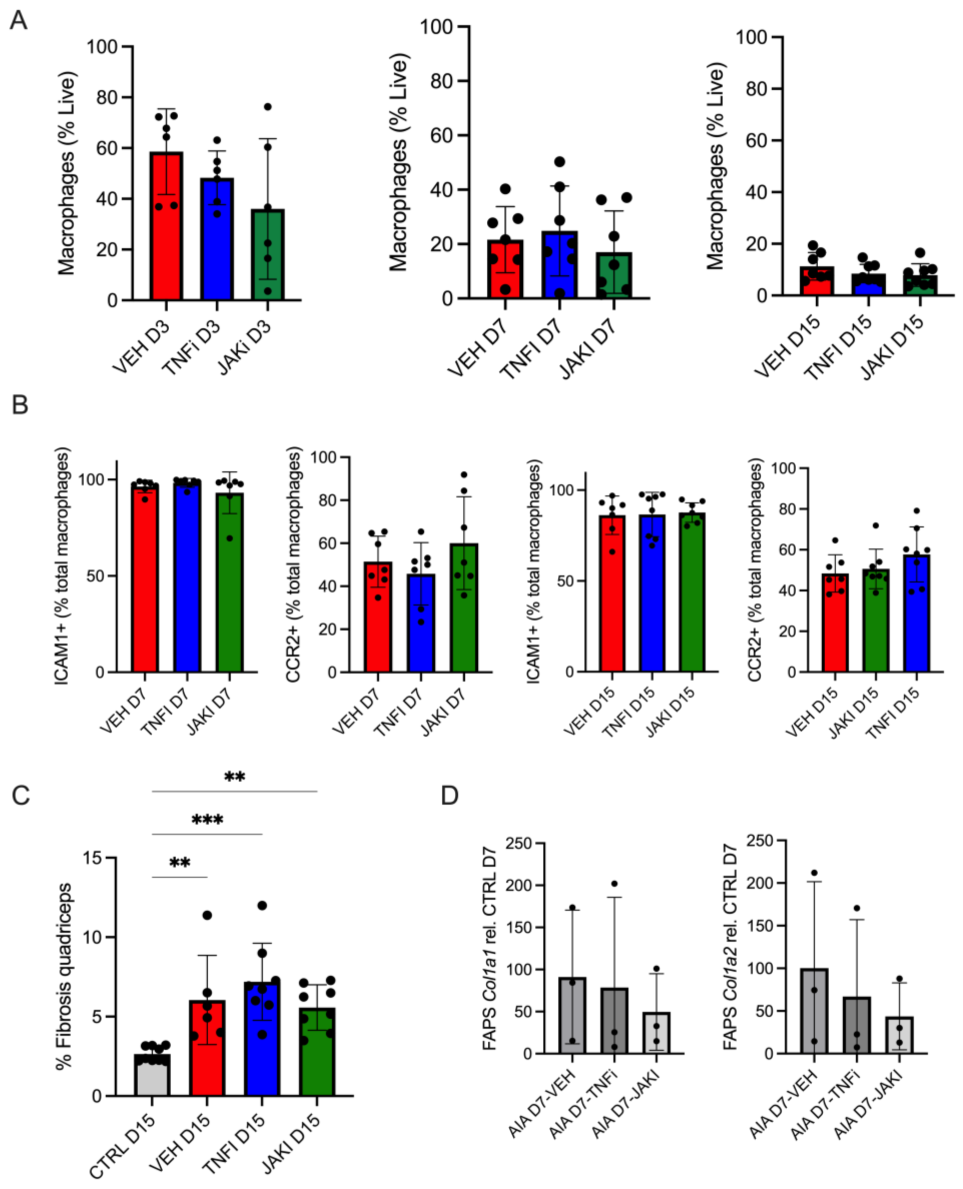

Supplementary Figure 4. Neither TNF inhibition nor JAK inhibition significantly affected periarticular myositis or muscle fibrosis in inflammatory arthritis. (A) The proportion of macrophages in periarticular muscle was equivalent between treatment groups (*n* = 6-8 per group). (B) The proportion of ICAM1+ and CCR2+ macrophages was equivalent between treatment groups. (D) FAPs were isolated from periarticular quadriceps muscles of AIA and control mice using fluorescence activated cell sorting and analysed for collagen type I gene expression. Each data point represents muscles pooled from 2-3 mice.

Mean ± SD presented. * $p < 0.05$; ** $p < 0.01$; *** $p < 0.001$; **** $p < 0.0001$.

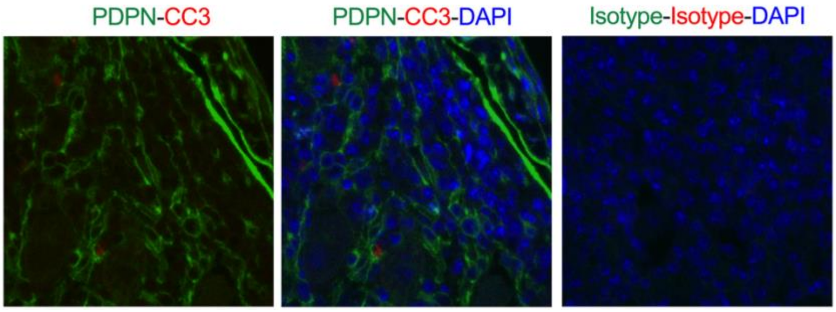

Supplementary Figure 5. Immunofluorescence staining of AIA muscle collected day 3 post arthritis induction with isotype control antibodies for anti-CC3 and anti-PDPN.

Supplementary Table 1

qPCR primers

Gene	Species	Forward	Reverse
GAPDH	Mouse	CCAGGTTGTCTCCTGCGACTT	CCTGTTGCTGTAGCCGTATTCA
TGFβ	Mouse	AGACATTCGGGAAGCAGTGC	TTCCGTCTCCTTGGTTCAGC
TNF	Mouse	ACTGAACTTCGGGGTGATCG	TGATCTGAGTGTGAGGGTCTGG
IL1β	Mouse	GCTACCTGTGTCTTTCCCGT	ATCTCGGAGCCTGTAGTGC
Col1a1	Mouse	GAGCGGAGAGTACTGGATCG	GCTTCTTTTCCTTGGGGTTC
Col1a2	Mouse	CAGCGAAGAACTCATACAGC	GACACCCCTTCTACGTTGT

Supplementary Table 2

Antibodies

Fluorochrome	Antigen	Reactivity/Host	Clone	Source	Catalogue	RRID	Dilution
APC-Cy7	CD45	Mouse/Rat	30-F11	BD Biosciences	561037	AB_10563075	1/400
BV785	CD11b	Mouse/Rat	M1/70	Biolegend	101243	AB_2561373	1/400
PE-Cy7	Ly6C	Mouse/Rat	AL-21	BD Biosciences	560593	AB_1727557	1/400
BV711	Ly6G	Mouse/Rat	1A8	BD Biosciences	563979	AB_2738520	1/400
BV421	CD64	Mouse/Mouse	X54-5/7.1	Biolegend	139309	AB_2562694	1/400
APC	ICAM1	Mouse/Rat	YN1/1.7.4	Biolegend	116120	AB_10613645	1/400
BV510	MHC II	Mouse/Rat	M5/114.15 .2	Biolegend	107636	AB_2561397	1/400
Biotin	LYVE1	Mouse/Rat	ALY7	Invitrogen	13-0443-82	AB_1724157	1/400
PE	CCR2	Mouse/Rat	475301	R&D	FAB5538P	AB_10718414	1/50
BV421	SCA1	Mouse/Rat	D7	Biolegend	108128	AB_2563064	1/400
PE-Cy7	CD31	Mouse/Rat	390	Biolegend	102418	AB_830757	1/800
APC	PDGFRα	Mouse/Rat	APA5	Invitrogen	135908	AB_2043970	1/400
PE	Podoplanin	Mouse/Rat	390	Biolegend	102408	AB_312903	1/400
AF700	α-7 integrin	Mouse/Rat	ITGA7	Invitrogen	MA5-23607	AB_2607369	1/200
Unconjugated	Fc Block	Mouse/Rat	2.4G2	BD Biosciences	553141	AB_394656	1/400
Unconjugated	Podoplanin	Mouse/Syrian Hamster	8.1.1	Biolegend	127402	AB_1089187	1/200
Unconjugated	CC3	Mouse/Rabbit	Polyclonal	R&D	AF835	AB_2243952	1/500
Unconjugated	CD45	Human/Mouse	2B11 + PD7/26	Dako	M0701	AB_2314143	1/1000
Unconjugated	CD68	Human/Mouse	PG-M1	Dako	M0876	AB_2074844	1/100

Supplementary Table 3. Patient characteristics.

Age, Gender	Relevant diagnoses	Immunomodulatory therapy*	Inflammatory markers*	Indication for PET/CT	Active joints on PET/CT	Degree of joint activity on PET/CT	PET avidity in peri-articular muscles	
							**Definite**	**Possible**
78, F	RA<br>*serology unknown*<br>Non-small cell lung cancer.	Nil	CRP 6.4mg/L	Lung nodule	Shoulders, right elbow, wrists, MCP joints	Moderate	No	Yes<br>Bilateral subscapularis
55, F	RA<br>*anti-CCP+, RF+*	MTX 20mg weekly, PNL 10mg daily, HCQ 200mg daily<br><br>RTX 1g administered 1 month prior to PET/CT	CRP 12.1mg/L<br>ESR 32mm/hr	Lung nodule in the context of previous renal cell carcinoma	Knees	Moderate	No	No
78, M	RA<br>*serology unknown*<br>Lung adenocarcinoma	MTX 10mg weekly, PNL 15mg daily	CRP 4.2mg/L<br>ESR 45 mm/hr	Lung lesion	Shoulders	Mild-moderate	Yes<br>Subscapularis	Yes<br>Supraspinatus
33, F	RA<br>*anti-CCP+, RF+*<br>Urinary tract infection	MTX 20mg weekly, PNL 37.5mg daily, HCQ 400mg daily	CRP 98.6mg/L<br>ESR > 100 mm/hr	PUO and lymphadenopathy	Shoulders, wrists, knees, ankles	Intense	Yes<br>Subscapularis	No
73, F	Inflammatory polyarthritis<br>*ANA+, anti-Ro52+*	Nil	CRP 7.6mg/L<br>ESR 75mm/hr	Systemic illness, possible vasculitis	Shoulders, knees	Mild-moderate	No	No

*At time of PET/CT

ANA, anti-nuclear antibodies; CCP, cyclic citrullinated peptide; CRP, C-reactive protein; ESR, erythrocyte sedimentation rate; HCQ, hydroxychloroquine; MCP, metacarpal phalangeal; MTX, methotrexate; PET, positron emission tomography; PNL, prednisolone; PUO, pyrexia of unknown origin; RA, rheumatoid arthritis; RF, rheumatoid factor; RTX, rituximab.
